# Functional assembly of tropical montane tree islands in the Atlantic Forest is shaped by stress tolerance, bamboo presence, and facilitation

**DOI:** 10.1002/ece3.7824

**Published:** 2021-07-02

**Authors:** Tina Christmann, Bruno H. P. Rosado, Guillaume Delhaye, Ilaíne S. Matos, Julia S. Drummond, Helena L. Roland, Yan C. Moraes, Imma Oliveras Menor

**Affiliations:** ^1^ School of Geography and the Environment University of Oxford Oxford UK; ^2^ Laboratório de Ecologia Vegetal Department of Ecology Universidade do Estado do Rio de Janeiro Rio de Janeiro Brazil; ^3^ Macrosystems Ecology Laboratory University of California Berkeley Berkeley CA USA

**Keywords:** biotic interactions, *Campos de Altitude*, CSR strategy, environmental filtering, tropical montane grasslands, woody communities

## Abstract

**Aims:**

Amidst the *Campos de Altitude* (Highland Grasslands) in the Brazilian Atlantic Forest, woody communities grow either clustered in tree islands or interspersed within the herbaceous matrix. The functional ecology, diversity, and biotic processes shaping these plant communities are largely unstudied. We characterized the functional assembly and diversity of these tropical montane woody communities and investigated how they fit within Grime's CSR (C—competitor, S—stress‐tolerant, R—ruderal) scheme, what functional trade‐offs they exhibit, and how traits and functional diversity vary in response to bamboo presence/absence.

**Methods:**

To characterize the functional composition of the community, we sampled five leaf traits and wood density along transects covering the woody communities both inside tree islands and outside (i.e., isolated woody plants in the grasslands community). Then, we used Mann–Whitney test, *t* test, and variation partitioning to determine the effects of inside versus outside tree island and bamboo presence on community‐weighted means, woody species diversity, and functional diversity.

**Results:**

We found a general SC/S strategy with drought‐related functional trade‐offs. Woody plants in tree islands had more acquisitive traits than those within the grasslands. Trait variation was mostly taxonomically than spatially driven, and species composition varied between inside and outside tree islands. Leaf thickness, wood density, and foliar water uptake were unrelated to CSR strategies, suggesting independent trait dimensions and multiple drought‐coping strategies within the predominant S strategy. Islands with bamboo presence showed lower Simpson diversity, lower functional dispersion, lower foliar water uptake, and greater leaf thickness than in tree islands without bamboo.

**Conclusions:**

The observed functional assembly hints toward large‐scale environmental abiotic filtering shaping a stress‐tolerant community strategy, and small‐scale biotic interactions driving small‐scale trait variation. We recommend experimental studies with fire, facilitation treatments, ecophysiological and recruitment traits to elucidate on future tree island expansion and community response to climate change.

## Graphical abstract

We assessed functional traits of woody communities in tree islands and grasslands in the montane Atlantic forest. The woody communities are stress‐tolerant and respond to facilitation and bamboo‐encroached tree islands show reduced diversity. This has implications for tree island expansion and their vulnerability to climate change.

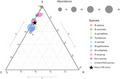

## 1 INTRODUCTION

Tropical montane ecosystems harbor exceptionally high biodiversity, due to a combination of unique abiotic conditions and isolation (Aparecido et al., 2018; Safford, 1999a). They provide important ecosystem services worldwide (Aparecido et al., 2018) but are threatened by global environmental changes (Assis & de Mattos, 2016; Oliveira et al., 2014), such as drought caused by reductions in fog exposure (Eller et al., 2016). Tropical mountain ecosystems in the Brazilian Atlantic Forest are found above 1,500 m asl, where diverse microenvironmental conditions give rise to various vegetation formations from grasslands and shrublands to bogs (Christmann & Oliveras, 2020). These ecosystems are called *Campos de Altitude* and are characterized by grass‐dominated mountaintop formations restricted to the highest summits of the southeastern Brazilian Highlands (Safford, 1999a). Abiotic factors such as recurring drought, frost, and rainfall seasonality (Safford, 1999b), as well as temporary disturbances (fire, mechanical damage), shape the physiognomy and composition of the vegetation in the *Campos de Altitude* (Assis & de Mattos, 2016), which shows a large functional and ecophysiological diversity (Duarte et al., 2005; Matos et al., 2020; Scarano et al., 2001).

The grassland communities of the *Campos de Altitude* have been relatively well characterized (Assis & de Mattos, 2016; Matos et al., 2020; Scarano, 2009). However, the woody communities, occurring either in tree islands or as interspersed individuals within the grasslands, have been overlooked. Tree islands are “clusters of two or more trees surrounded by a dissimilar vegetation type” and are characteristic features in alpine ecotones (Resler & Stine, 2009). In mountain regions, tree islands often occur as part of the tree line ecotone where small‐scale topographic features improve edapho‐climatic conditions for tree growth, while between tree islands, seedling mortality limits tree establishment (Harsch & Bader, 2011). Trees in islands persist due to positive feedback mechanisms caused by microclimatic facilitation inside islands that ameliorates and reduces exposure to harsh conditions, even promoting tree infilling and tree island expansion (Albertsen et al., 2014; Resler & Stine, 2009). The edges of tree islands can harbor safe sites for grassland species, as reported for tree islands at the forest–tundra ecotone (Albertsen et al., 2014).

The presence of these tree islands in the *Campos de Altitude* could be explained by two hypotheses. First, tree islands could be remnants from a time of absence of fire in these areas; an increase in fire frequency in the last centuries might have restricted montane forest expansion, notably in the mountains of Itatiaia National Park (Behling et al., 2020). Second, those islands could be a result of woody encroachment either due to recent climate change or due to wetter and warmer periods since the late mid‐Holocene (Behling et al., 2020). In both cases, initial small tree islands could have provided microclimatic shelter and facilitated further tree establishment, as observed in temperate alpine regions (Resler & Stine, 2009).

The assembly of these woody communities can be studied through the lens of community ecology. Abiotic filters and biotic interactions select a subset of species able to survive and coexist in a given environment (reviewed in Cadotte & Tucker, 2017; Kraft et al., 2015). The two main biotic processes acting at the community level are competition and facilitation (see Brooker et al., 2008). Competition refers to negative interactions between co‐occurring plants and can negatively impact the establishment and growth of the woody community and the tree islands. On the contrary, facilitation is a positive interaction mediated through amelioration in the physical environment and has been described as an important process in high altitude ecosystems, where many species are at their physiological limits of survival (Callaway et al., 2002).

The study of functional traits (sensu Violle et al., 2007) has become one of the most promising ways to understand the assembly of plant communities and their response to environmental change (Funk et al., 2017; Lavorel & Garnier, 2002; McGill et al., 2006). Because of developmental, evolutionary, and environmental constraints, functional traits exhibit different levels of coordination and trade‐offs, leading to sets of traits related to the same function (Chapin et al., 1993; Delhaye et al., 2020; Wright et al., 2004). At the global scale, the leaf economic spectrum (Wright et al., 2004), the wood economic spectrum (Chave et al., 2009), and the whole plant economics spectrum (Reich, 2014) have been widely used to classify species along a continuum from fast growth and high resource acquisition to slow growth and high resource conservation. Another classification of strategies based on those trade‐offs is the CSR strategy (Grime, 1977), which categorizes plants as competitor (C), stress‐tolerant (S), or ruderal (R). This can be done based on three easily measurable leaf traits that represent extremes of the leaf economic (specific leaf area and leaf dry matter content) and leaf size spectrum (leaf area, LA) and which have been found to represent a broad range of plant functioning (Grime, 1977). By comparing values of these three leaf traits against a globally calibrated dataset, individual‐based CSR percentages can be extracted (Pierce et al., 2017).

CSR analysis can help predict how species will respond to changes in biogeochemical cycles, climate, and land‐use variation (Pierce et al., 2017) and has proven successful to investigate many ecological processes in a variety of environments (Li & Shipley, 2017; Pierce et al., 2017; Rosado & de Mattos, 2017), including the grassland community of the *Campos de Altitude* (Matos et al., 2020). In the *Campos de Altitude,* abiotic factors such as recurring drought and frost (Safford, 1999a) shape the grassland community, pushing species toward stress‐tolerant ecological strategies (Matos et al., 2020).

However, the species in these communities can differ in relation to ecophysiological integrative traits (Matos et al., 2020). One such trait that has been shown to be key to survival in tropical mountain environments is foliar water uptake, the ability of plants to absorb water through their leave (Eller et al., 2016; Matos et al., 2020). Integrative traits reflect the results of the combinations of multiple functional traits and are more responsive to environmental factors (Rosado & de Mattos, 2017). A multivariate analysis of morphological and physiological traits of the grassland community by Matos et al. (2020) showed a first trait dimension of drought resistance (via high LDMC and high stem specific density) versus resilience (via high resprout ability and high leaf water potential at turgor loss point). Thereby, stress‐tolerant plants (S) showed traits associated with drought resistance (e.g., high LDMC and high stem specific density) while competitive‐ruderal (CR) plants showed more acquisitive traits and drought escape through resprouting ability and large seed size. The second trait dimension was underlined by a trade‐off between water storage (via succulence) versus water absorption (via foliar water uptake). CR‐species showed a tendency toward water absorption, while species with a mixture of stress tolerance and competitiveness (CS) exhibited water storage traits (Matos et al., 2020). However, the ecological strategies and ecophysiology of woody communities above the tree line and particularly in the tree islands are still unknown.

Regarding the effect of biotic interactions, because of their physiognomy, tree islands could provide shelter through facilitation and favor tree infilling (Albertsen et al., 2014; Mendoza‐Hernández et al., 2013; Resler & Stine, 2009). On the contrary, strong competitive interactions could be expected from the presence of the dwarf bamboo species *Chusquea pinifolia* (Poaceae) in many tree islands. *C*. *pinifolia* is a clear stress‐tolerant (S) species and many of its functional traits have been studied by Matos et al. (2020). *C*. *pinifolia* is abundant and native in the *Campos de Altitude* (Safford, 1999a) and occurs both within the grasslands and inside the tree islands. Some species of *Chusquea* have been shown to turn into aggressive colonizers under disturbance, modifying vegetation structure, soil and nutrient properties, and decreasing woody species diversity elsewhere, even within their native ranges (Pagad, 2016). The presence of *C*. *pinifolia* could have a negative effect on the ecology and functional composition of the tree islands, as shown in Amazonian bamboo forests (Fadrique et al., 2021).

Here, we characterized the functional composition of 10 tree islands and their neighboring woody communities within the grassland, using six traits related to the plant economics spectrum and water use. We also evaluated the effect of facilitation and bamboo presence/absence on the community trait composition. Our overarching hypothesis was that environmental abiotic conditions would shape the woody communities by selecting for a set of conservative traits and a stress‐tolerant strategy, with bamboo presence influencing trait assembly and reducing the species diversity of tree islands. Specifically, we expected (a) a strong S‐selection in the woody communities, supported by a set of conservative functional trait values (e.g., high LDMC, high wood density, and low SLA) and trade‐offs due to harsh abiotic conditions (e.g., drought, fire, and frost); (b) less conservative trait values of woody plants inside tree islands compared to woody plants within the grasslands due to facilitative processes; (c) differences in species composition and traits associated with stress tolerance strategy (low SLA, high FWU, and high WD) in islands where bamboo is present in comparison with bamboo‐free islands. We further expected a decrease in woody taxonomic and functional diversity due to a convergence to a more water‐conservative strategy due to competitive effects in response to bamboo presence.

## 2 MATERIALS AND METHODS

### 2.1 Study area

The study was conducted in August 2019 in woody communities in the grasslands “*Campos de Altitude*” of the Itatiaia National Park in the Brazilian Atlantic Forest. The *Campos de Altitude* are found in Rio de Janeiro state (22°22′37″S 44°42′28″W) above the tree line, which lies between 1,800 and 2,300 m asl, and are cool‐humid, grass‐dominated mountaintop formations restricted to the highest summits of the southeastern Brazilian Highlands. Soils in the *Campos de Altitude* vary locally depending on hydrology and topography, but are generally moderately fertile, with dark‐humic upper horizons and argillic‐podzolized lower horizons across the Altiplano (Safford, 1999a). The climate in Itatiaia has subtropical and temperate influences with a pronounced three‐month dry season (Jun‐Ago), when the precipitation is <50 mm/month and totals 77 mm for these three months (i.e., 4.8% of total annual precipitation; Segadas‐Vianna & Dau, 1961; Koeppen‐Geiger Cwa). Mean temperature is 14.4°C and yearly precipitation is between 2,000 and 2,200 mm. Frost occurs on average 56 days per year and fog 218 days per year (Safford, 1999a). Fires recur yearly, most of which are anthropogenic (Aximoff & de Carvalho Rodrigues, 2011).

### 2.2 Data collection

Since the tree islands are relatively rare azonal formations, we selected 10 tree islands within the *Campos de Altitude* Altiplano where (a) we could lay a transect from south to north without interfering with topographic features or another tree island, (b) it was possible to safely access them, and (c) the tree islands were larger than the transect length.

**FIGURE 1:**
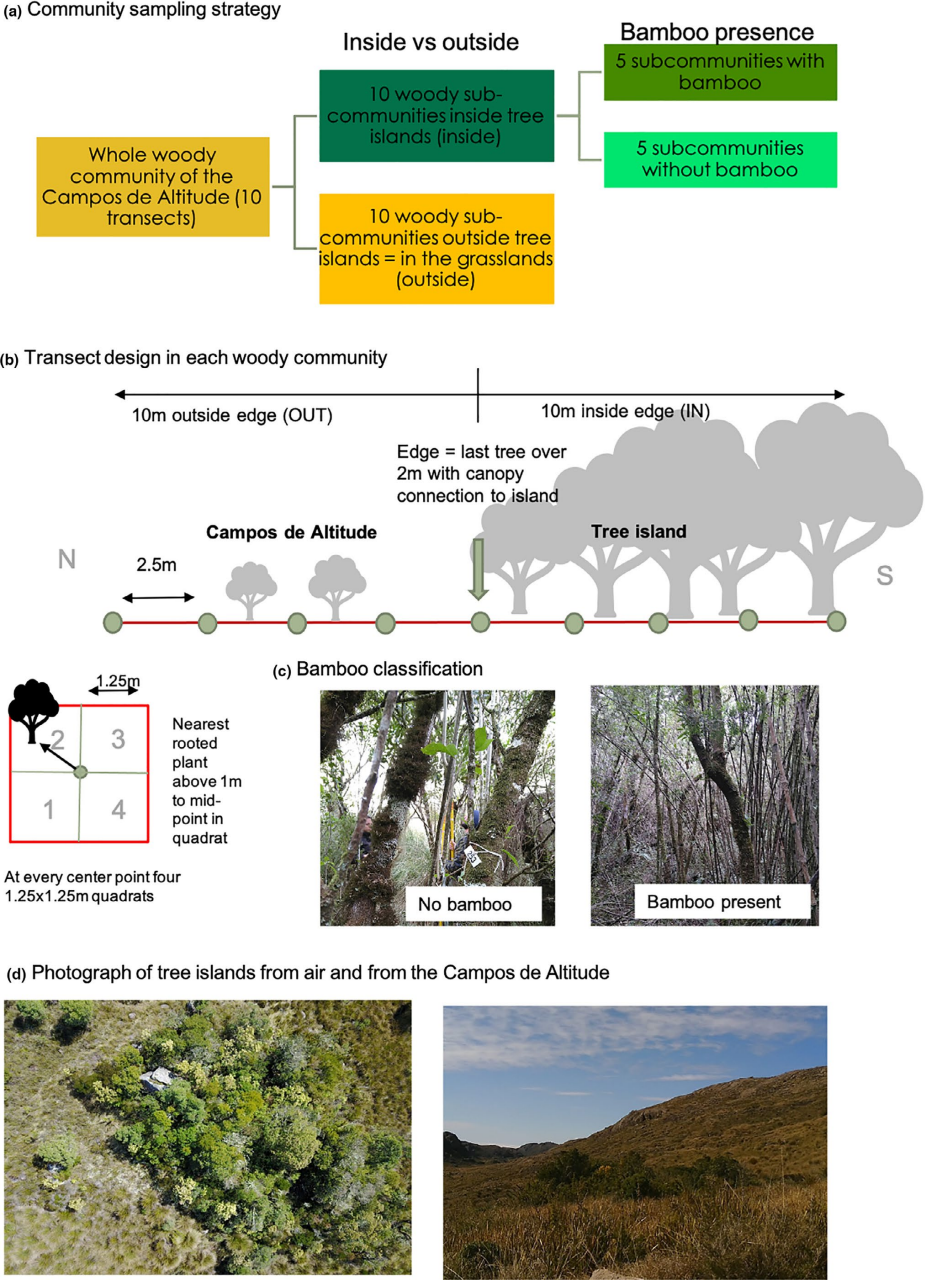
(a) Community sampling design; (b) sampling along transects through the tree islands; (c) example of the inside of an island with bamboo presence and without bamboo; and (d) two photographs of the tree islands, from air (photograph by Y. Moraes) and from the *Campos de Altitude* (photograph by T. Christmann)

Ten woody communities were assessed through 20 m transects covering 10m inside the tree island (hereafter “‘inside”) and 10m outside of the edge of the island within the grasslands (hereafter “outside”), all maintaining a south–north aspect (Figure 1b,d, Table S1). Therefore, there were 10 “inside” subcommunities and 10 “outside” subcommunities (Figure 1a). The edge of the tree island was defined as the last woody species higher than >2 m with a canopy overlap with the trees in the tree island. Mean canopy height inside tree islands was 3.88 m and outside 1.21 m (further information on vegetation structure in each tree island in Table S1). Sampling of woody communities was done with a point‐centered quarter method every 2.5 m. The closest rooted individual to the center point taller than 1m within each 1.25 × 1.25 m quadrant was sampled and tagged. If the closest individual was outside the quadrant, it was regarded as empty. Minimum distance between islands was 100 m, maximum distance 3.5 km, and size of tree islands ranged from 0.11 to 1.48 ha (Supplementary S1). While the distance of 10 m into the tree islands did not always correspond to the most internal location of the tree islands due to the varying sizes and shapes, we believe that edge effects occur throughout the tree islands and that the 10 m transects are broadly representative for the whole woody community inside tree islands.

Woody communities inside tree islands were classified as showing the presence of bamboo (“b”, 5 subcommunities) or no presence of bamboo (“n”, 5 subcommunities; Figure 1a), as they were overgrown by either bamboo or bamboo free (Figure 1c, Table S1).

We sampled traits on 188 individuals, located along 88 sampling points. Leaf dry matter content (LDMC [mg/g]), leaf area (LA [cm^2^]), specific leaf area (SLA [cm^2^/g]), and leaf thickness (LT [mm]) were measured on five intact fully developed sun leaves per individual. We measured wood density (WD [g/cm^3^]) on a piece of twig wood, instead of trunk wood as removing parts of the main stem would have caused large damage to the woody plants. All trait measurements followed Pérez‐Harguindeguy et al. (2013) (see Methods S1 for exact measurement procedures). All sampled species were evergreen.

Foliar water uptake (FWU, [%]) was measured based on the protocol of Limm et al. (2009) on three mature and healthy leaves per individual and calculated as the percentage increment in leaf water content after submersion (see Methods S2). FWU was then averaged across three leaves for each sampled plant to obtain a mean value.

Since ecosystem functions are largely determined by the abundant and dominant species (Avolio et al., 2019; Grime, 1998) for analysis comparing variation of traits or strategy between and within species, we focused on eight dominant species (Table S2), which comprised 80% of all sampled woody individuals.

### 2.3 Data analysis

Data processing was carried out in Microsoft Excel^®^ and data analysis in R (version 4.0.2) using the R studio interface (Version 1.2.1335).

#### 2.3.1 CSR strategy and trade‐offs

To test if the woody community exhibits a strong S strategy, CSR values were calculated using the globally calibrated ‘StrateFy’ tool in Excel (Pierce et al., 2017) which uses a principal component analysis (PCA) on three leaf functional traits to assign a percentage value for C, S, and R strategy to each sample. We calculated community‐weighted means (CWM) of traits and CSR strategies for the entire community (see Methods S3), as well as mean trait and CSR values for the eight “dominant” species. We tested for monotonic relationships between traits using Spearman's rank correlations on raw data. Comparison between inside and outside tree islands.

#### 2.3.2 Inside versus outside tree islands

To investigate differences in assembly process between communities inside and outside the tree islands, we compared the CWM values with a Mann–Whitney test, because response variables were not normally distributed, and no transformation resulted in normality. We further compared woody species richness and Simpson index (measure of species taxonomic diversity) between communities inside and outside tree islands with Mann–Whitney test and calculated Sørensen similarity index (R package “vegan”, Oksanen et al., 2019) to assess species similarity. We performed variation partitioning (R package “lme4”, Bates et al., 2015) to examine trait variation in response to species identity (24 species), inside versus outside tree islands and in response to differences between the 10 woody communities (i.e., the plots) with a linear mixed effect model, similarly to Oliveras et al. (2020) (Methods S4).

#### 2.3.3 Influence of bamboo on traits and diversity of tree islands

Difference in CWM of traits and CSR strategies between the five tree islands subcommunities with bamboo presence and the five subcommunities without bamboo presence was tested with Mann–Whitney test.

Sørensen similarity index and PERMANOVA (R package “vegan”, Oksanen et al., 2019) were calculated to compare differences in species abundances and composition between tree islands with and without bamboo. To assess differences in taxonomic and functional diversity between bamboo and non‐bamboo tree islands, woody species richness, Simpson index, and functional dispersion were calculated for inside each tree island subcommunity (10 inside subcommunities, of which 5 with bamboo presence and 5 without) as response variables. We calculated the functional dispersion index (FDis) for the six traits (SLA, LA, LDMC, WD, LT, and FWU) with the R package “FD” (Laliberté et al., 2015) since it can be computed from any distance or dissimilarity measure, can handle any number and type of traits (including more traits than species, which was the case in some of the tree islands), and is not strongly influenced by outliers (Laliberté & Legendre, 2010). The FDis index is by construction unaffected by species richness (Laliberté et al., 2015) which varied from 3–7 species between the tree islands. Since Simpson index and FDis were not normally distributed, differences between tree island subcommunities with or without bamboo were tested with Mann‐–Whitney test, while differences in species richness were tested with *t* test.

## 3 RESULTS

### 3.1 CSR strategy, functional assembly, and trade‐offs

Across the whole woody communities, we recorded a total of 24 woody species (Table S4.). Eight of those species (Table S2) comprised exactly 80% of all individuals and were considered as “dominant” species in further analysis. *Myrsine gardneriana* was the most abundant species (27% relative frequency), followed by *Baccharis stylosa* (13.5% relative frequency), *Myrsine umbellata, Pleroma trinervia,* and *Archibaccharis serratifolia* (all 8%–10% relative frequency; Table S2).

**FIGURE 2:**
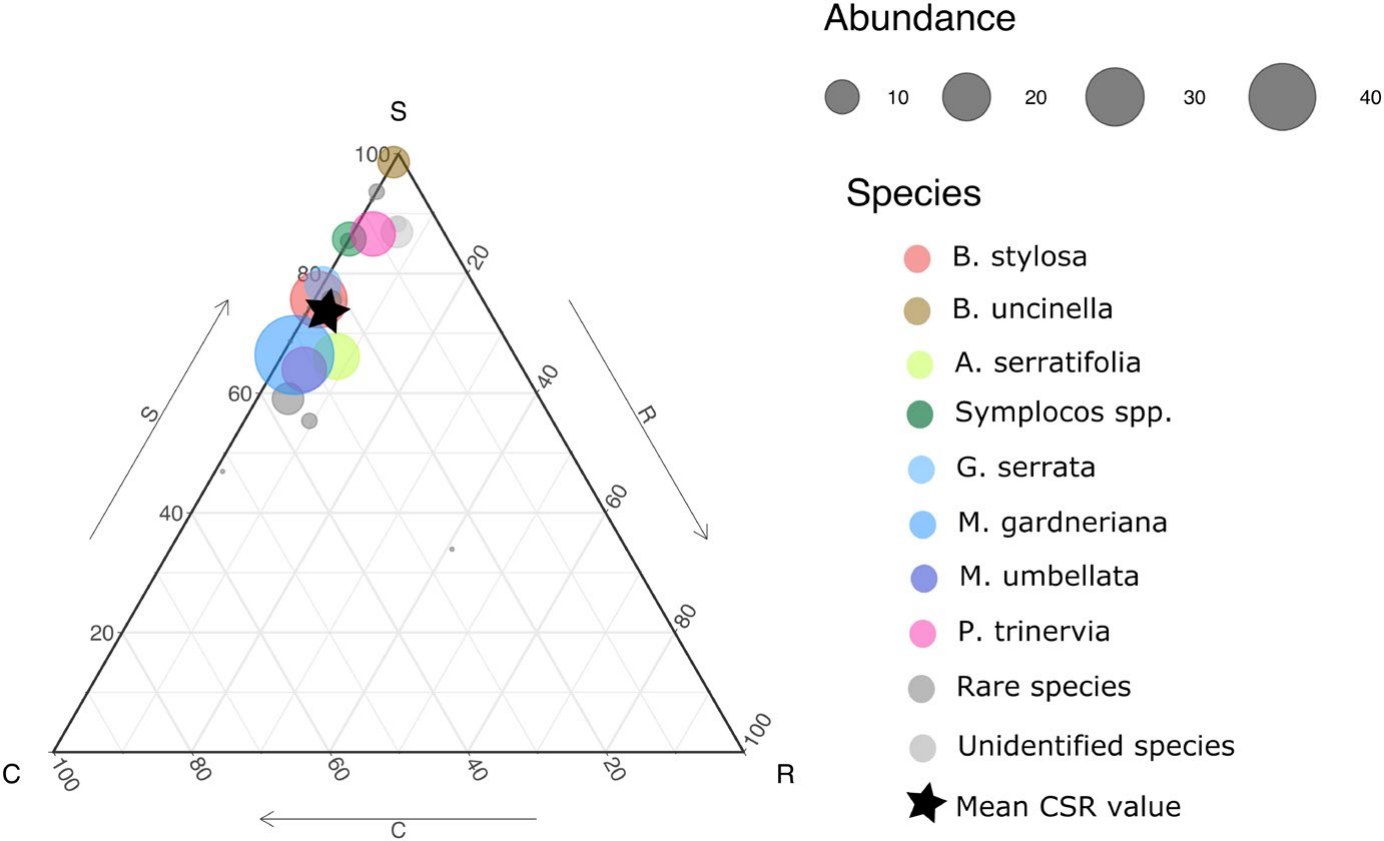
CSR composition for all species in the woody communities with species scales by their abundance (number of individuals). The eight dominant species (*Baccharis stylosa*, *Baccharis uncinella*, *Archibaccharis serratifolia*, Symplocos spp, *Gaultheria serrata*, *Myrsine gardneriana*, *Myrsine umbellata*, and *Pleroma trinervia*) are depicted in colors, the rare species in dark gray and the unidentified morphospecies in light gray. CWM of CSR strategy across the entire community is indicated by a star

The average vegetation CSR strategy of the woody community was stress‐tolerant (CWM of C:22.78%, S:75.09%, and R:2.13%; Figure 2; Table S2). CSR strategies differed significantly between the eight dominant species (Figure S1). Mean CWM values for SLA, LA, and LDMC across the woody communities were 17.2 cm^2^/g, 10.6 cm^2^, and 390 mg/g respectively, with *A*. *serratifolia* showing the highest average SLA (23.82 cm^2^/g), *M*. *gardneriana* the highest average LA (18.46 cm^2^), and *Baccharis uncinella* the highest LDMC (0.5 g/cm^3^; Table S2). Mean CWM of LT was 0.31 mm, mean WD 0.88 g/cm^3^, and mean FWU 16.4%, with the highest FWU (60.36%) observed in *B*. *uncinella* and the highest WD for the morphospecies of the genus *Symplocos* (1.09 g/cm^3^; Table S2).

The Spearman correlation (Table 1) showed that SLA was negatively correlated with both LDMC (*ρ* = −0.66, *p* < .01) and LT (*ρ* = −0.59, *p* < .01). FWU was weakly and negatively correlated with LT (*ρ* = −0.31, *p* < .1), yet uncorrelated with all other traits, as well as to CSR percentages (CSR%). CSR% were further uncorrelated with t LT, WD, and FWU.

**TABLE 1:** Spearman correlation table

	SLA	LA	LDMC	LT	WD	FWU	C	S
LA	−0.03							
LDMC	**−0.66** **	−0.24						
LT	**−0.59** **	0.11	0.19					
WD	−0.31	0.13	0.12	0.23				
FWU	0.25	−0.3	0.06	**−0.31** *	−0.13			
C	0.08	**0.93** **	−0.46*	0.12	0.11	−0.31		
S	−0.32	**−0.84** **	**0.61** **	0.03	−0.05	0.18	**−0.93** ***	
R	**0.84** **	0.01	**−0.53** **	**−0.50** **	−0.24	0.22	0.04	−0.33

Notes

The asterisks indicate the significance bold values.

* *p* < 0.1

** *p* < 0.01

*** *p* < 0.001.

### 3.2 Functional assembly inside versus outside the tree islands

**FIGURE 3:**
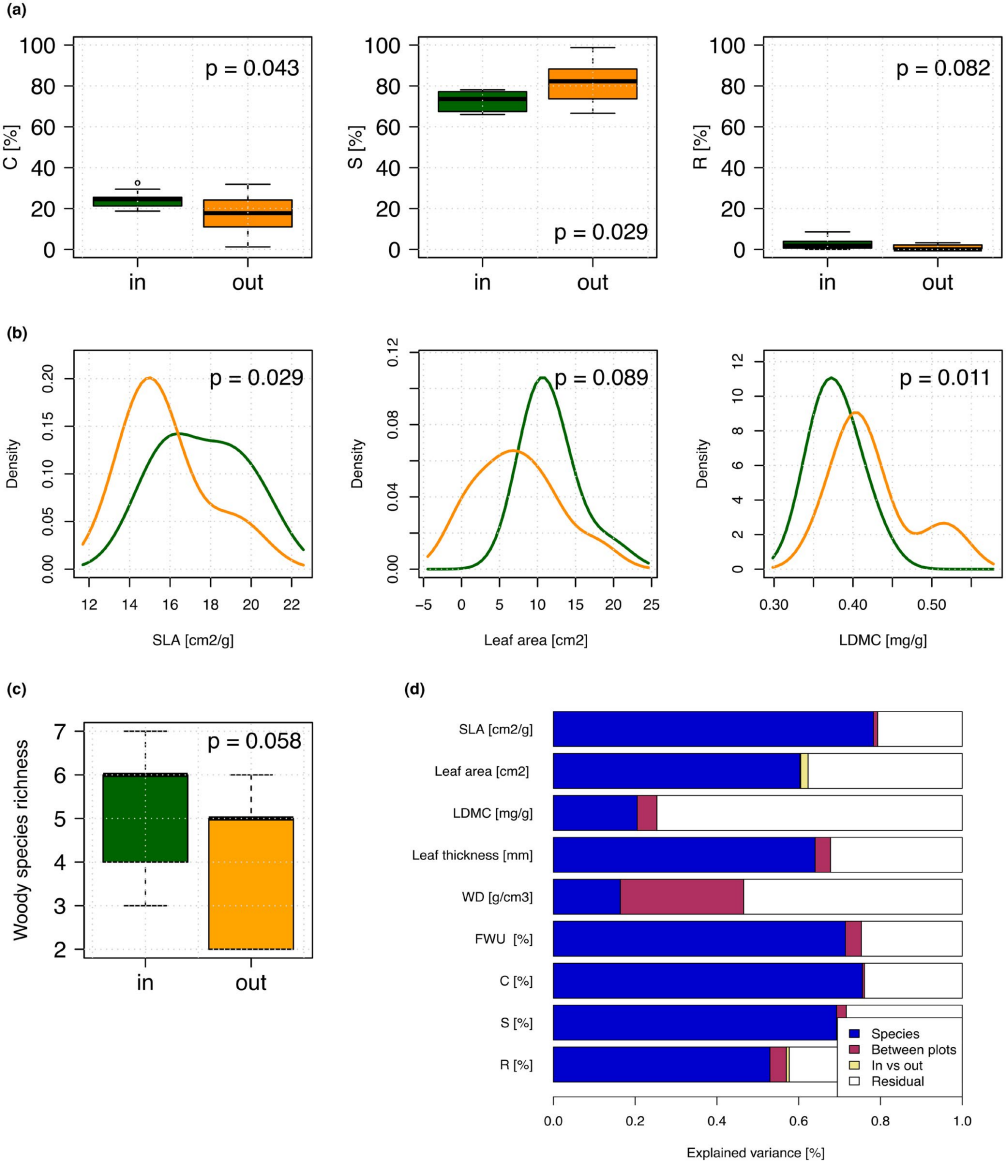
(a) Boxplot of variation for community‐weighted means of CSR strategy between communities inside (*n* = 10) and outside tree islands (*n* = 10); (b) density plots for significant variation in community‐weighted mean traits between the 10 communities inside (dark green) and outside (orange) the tree islands; (c) variation in woody species richness between the subcommunities inside and outside the tree islands; (d) variation partitioning for variation in traits and CSR strategy (number of total samples for each trait *n* = 178) between species (*n* = 24), plots (*n* = 10), and between inside versus outside (*n* = 2)

A total of 18 woody species occurred inside tree islands while 15 species occurred outside, and eight species were shared between inside and outside (Sørensen index = 0.55; Table S2). The average subcommunity inside a tree island had more woody species than the average subcommunity outside (5.4 vs. 4.1); however, the difference was below the significance level (Figure 3b).

Six of the “dominant” species occurred both inside and outside, while the “dominant” species *Gaultheria* *serrata* and *B*. *uncinella* occurred exclusively outside the tree islands. Species predominantly growing outside the tree islands (e.g., *P*. *trinervia*, *Symplocos* sp., *Baccharis uncinella*, and *G. serrata*) showed high S‐percentages, while species occurring both inside and outside the tree islands (e.g., *M*. *gardneriana*, *Baccharis stylosa*, *A*. *serratifolia,* and *M*. *umbellata*) had higher C‐percentages (Table S2).

S‐percentages were significantly higher, and C‐percentages were lower for woody plants within the grasslands in comparison with inside the tree islands (Figure 3a). Community‐weighted means of CSR strategy were C 24.72%: S 72.73%: R 32.55% for communities inside tree islands and C 16.38%: S 82.69%: R 0.93% outside tree islands (Table S3). SLA was significantly higher (*p* = .029) in tree islands, while LDMC was significantly higher (*p* = .011) in trees growing in the grasslands. Mean values for LA were higher in tree islands, albeit not significant (*p* = .089; Figure 3b). There was no difference in any of the other traits between woody plants growing inside and outside the tree islands. The variation partitioning showed that species explained 50%–80% of the variation for all traits except LDMC and WD, which showed larger residual variation. The effect of inside versus outside was negligible for all traits in the variation partitioning. Differences between plots (i.e., the 10 woody communities) explained a larger proportion of variation in all traits and CSR percentages than differences between inside and outside in all traits except LA (Figure 3d). Particularly, 30% of the variance in WD was explained by differences between plots (i.e., the 10 transects).

### 3.3 Influence of Bamboo on Traits and diversity of tree islands

**FIGURE 4:**
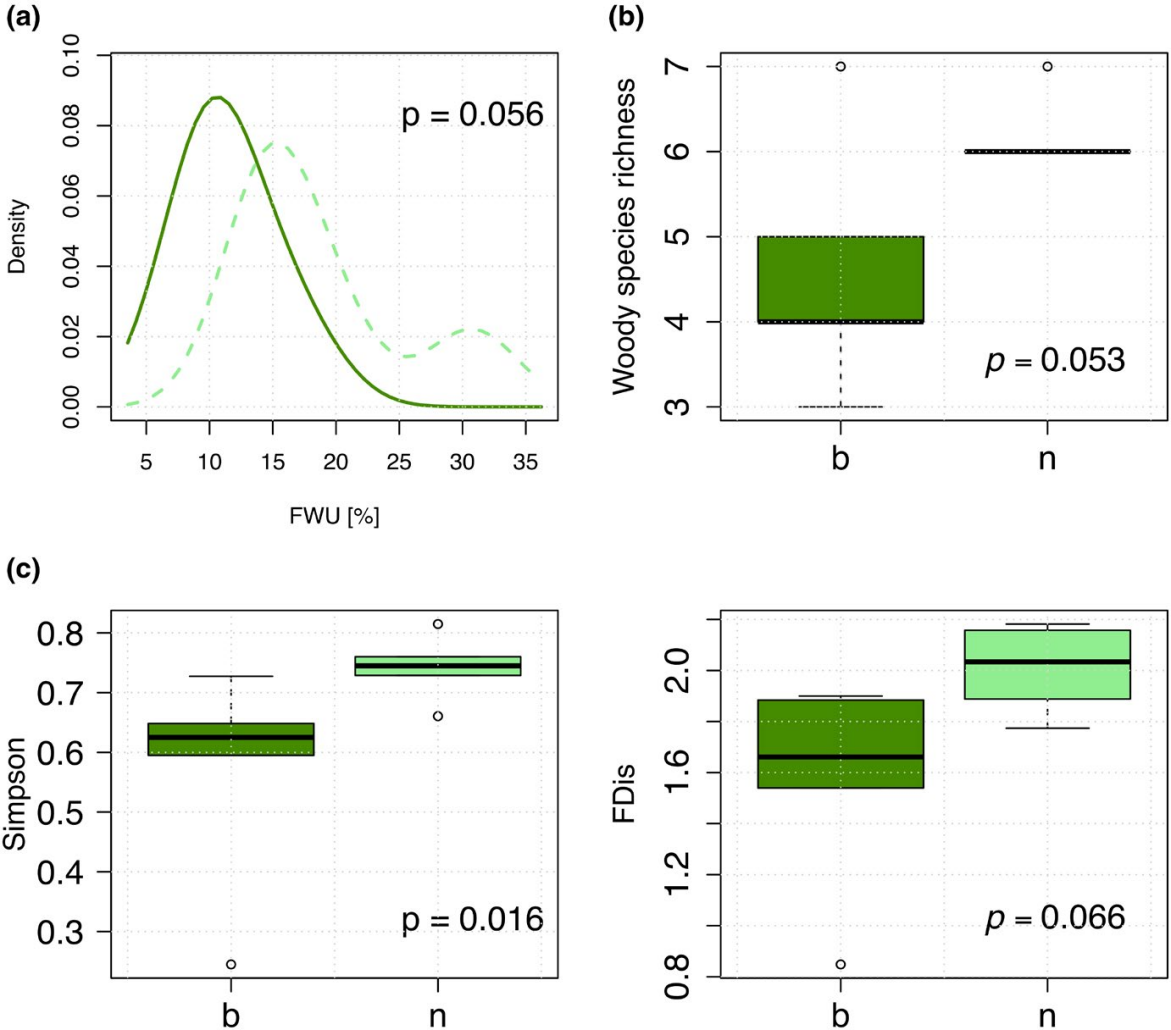
Variation of traits and diversity metrics in response to bamboo presence (a) density plot for FWU in bamboo and non‐bamboo islands, (b) boxplots for variation in species richness, and (c) diversity metrics (woody species richness, Simpson diversity, and functional dispersion) between tree island subcommunities with bamboo presence (*n* = 5) and without bamboo (*n* = 5)

A total of 18 species occurred across all the five islands with bamboo presence, compared to 16 species in islands without bamboo, and 10 species were shared between both (Sørensen similarity index of 0.59). PERMANOVA analysis showed no significant difference (*p* = .12) in species composition between islands with and without bamboo. Islands with bamboo presence had on average less species (*p* = .053) and a significantly lower Simpson index (*p* = .016) than bamboo‐free islands (Figure 4b). Woody species richness and FDis were uncorrelated (Spearman *ρ* = −0.14), showing that FDis was independent of species numbers and thus a good metric to compare functional diversity between islands. Means of FDis were lower in islands with bamboo than in bamboo‐free islands, albeit marginally non‐significant (1.56 vs. 2, *p* = .066; Figure 4c).

CSR strategies did not differ significantly between woody plants growing in islands with or without bamboos (Table S4), accordingly neither SLA, LA nor LDMC differed significantly. Woody plants occurring in islands with bamboo presence showed lower FWU means (Figure 4a) than in islands without bamboo, but again just below the significance level (11.523 vs. 15.05, *p* = .056; Table S4).

## 4 DISCUSSION

Woody plants from the Campos de Altitude show a convergence to a stress‐tolerant functional strategy compared to the global CSR space. Woody plants inside tree islands showed more acquisitive leaf traits and a dominant CS strategy, while plants outside were more conservative in resource use, exhibiting a dominant S strategy. Bamboo presence reduced Simpson diversity of woody communities, and contrary to our expectations, woody plants in communities with bamboo presence showed a trend toward lower foliar water uptake. We discuss these findings in connection to concepts of biotic interactions, abiotic filtering, and potential implications for the tree island ecosystems under climate change.

### 4.1 CSR strategy and trade‐offs in the woody community

Overall, the woody communities showed a similar CSR strategy to the tussock grasses and shrubs on the *Campos de Altitude* assessed by Matos et al. (2020). With the prevalent conservative SC/S strategy and the functional trait trade‐offs, the functional assembly of the woody communities is clearly a result of strong environmental filtering, such as the seasonally recurring droughts and frost in Itatiaia National Park (Safford, 1999b). A large proportion of the trait variation was driven by differences in species identity, which indicates that filtering across these woody communities is largely expressed through taxonomic variation, in accordance with other ecological gradients in the tropics (Oliveras et al., 2020). Our species list did not allow for analyses to test if this taxonomic variation could be explained by the shared evolutionary history of species (i.e., phylogeny). Further studies on the role of evolutionary trait patterns in the alpine Atlantic forest environment would shed light on this important topic.

Despite the convergence toward a stress‐tolerant strategy in the woody communities, traits not feeding into the CSR analysis (i.e., foliar water uptake, wood density, and leaf thickness) were independent from CSR strategies. The uncoupling of leaf thickness and wood density, as well as foliar water uptake from the CSR strategies, is in line with findings by Rosado and de Mattos (2017) and the twin filter model (Grime & Pierce, 2012). According to this model, co‐occurring species sharing the same overall CSR strategy can diverge in ecophysiological traits. While Matos et al. (2020) found three ecophysiological strategies in the grasses and shrubs of the *Campos de Altitude*, we here found two main ecophysiological strategies for woody plants. We observed S‐species with a conservative resource strategy and high foliar water uptake occurring predominantly outside the tree islands (*Baccharis uncinella*, *Pleroma trinervia*) and CS‐species with variable levels of foliar water uptake inside the tree islands (*Myrsine gardneriana*, *Myrsine umbellata*, and *Archibaccharis serratifolia*).

Wood density, a trait related to mechanical stability and hydraulic safety (Eller et al., 2018; van der Sande et al., 2019), showed variation between tree islands, perhaps due to different hydrological conditions or varying exposure to wind stress of the tree islands selected for specific stem wood densities. This might point toward the existence of different ecophysiological wood strategies related to xylem water transport between communities. Low wood density is associated with high transport efficiency but low hydraulic safety, while high wood density is connected to low transport abilities but higher hydraulic safety and lower vulnerability to embolism (Chave et al., 2009). This is in line with finding that multiple drought survival strategies often coexist (Pivovaroff et al., 2016; Rosado et al., 2013; Rosado & de Mattos, 2017). While we did not measure abiotic site conditions in the different islands, future assessment of hydrological and soil conditions will be needed to better link trait response to underlying environmental factors.

Within the whole dataset, we found a trade‐off between leaf thickness and foliar water uptake. This aligns with findings by Gotsch et al. (2015) who found lower foliar water uptake in species with thicker leaves and greater water storage capacity, while thin leaves enable higher foliar water uptake. This trade‐off was particularly visible in two of the dominant species outside the tree islands; *Baccharis uncinella* showed the highest levels of mean foliar water uptake (60%) and relatively thin leaves, while *Gaultheria* *serrata* showed the highest mean thickness coupled with the lowest foliar water uptake. This underlines two main types of drought response: thicker leaves could be associated with either a drought avoidance strategy in succulent species, where water is stored in leaf tissue (Ogburn & Edwards, 2010), or to a drought tolerance strategy in sclerophyllous species (Gullo & Salleo, 1988; Rhizopoulou & Psaras, 2003). This study shows that both drought‐coping mechanisms coexist in the woody community.

### 4.2 The functional assembly inside versus outside the tree island

We found higher competitiveness and lower stress tolerance inside tree islands compared to outside. This is further confirmed by more acquisitive single traits (higher specific leaf area, lower leaf dry matter content) inside the tree islands compared to outside. This could be either due to (a) higher need for competitive traits when co‐occurring with trees sharing similar requirements (e.g., light) inside the tree islands or (b) facilitative processes decreasing environmental stress, through microclimatic shelter (e.g., higher soil moisture and nutrient concentration) as previously observed in montane tree islands (Albertsen et al., 2014), subsequently enabling resource allocation toward more competitiveness and less stress tolerance.

Facilitation has been found to be stronger in resource‐limited or relatively “extreme” environments, such as deserts and alpine tundra, close to the physiological and altitudinal limits of a species, and in intermediate to high levels of climate stress and disturbance, where it allows species to expand into harsher conditions (Brooker et al., 2008). An interesting avenue for future research would be to investigate whether the species in the tree islands are close to their temperature and water availability niche extrema using species distribution modeling. This could show whether facilitation in the first place enables woody species, many of which prominent cloud forest species like *Myrsine gardneriana* and *Myrsine umbellata* (Pompeu et al., 2014), to exist far above the tree line. The woody species observed in our study might both benefit from facilitation but also provide facilitation; in forest–grassland transitions, in southern Brazil, *M*. *umbellata* for instance has been shown to promote seedling abundance and seedling species richness under its crown, making it a facilitator species (Mendoza‐Hernández et al., 2013).

We found comparatively low levels of foliar water uptake inside the tree island communities (CWM of FWU of 15.17%) compared to measurements of grass and shrub species in the *Campos de Altitude* (mean FWU of 73.6%) by Matos et al. (2020). On the one hand, this could be associated with differences in rooting depths, as species with shallow roots, like many grasses and shrubs, show higher capacity for FWU than species with deep roots which have better all‐year‐around water access (Cavallaro et al., 2020). On the other hand, this could be another proof of facilitation, since in the tree islands drought buffering due to sheltering effects could be at play, resulting in a lower need for alternative mechanisms of water acquisition.

Five limiting mechanisms have been hypothesized to operate in woody communities of alpine tree line ecotones: stress, disturbance, reproduction limitation, growth limitation, and carbon balance (Körner, 1998). Based on the observed functional assembly of stress tolerance, but differences in stress tolerance between inside and outside tree islands it is likely that the “stress hypothesis” primarily applies to these communities. While prevailing drought and frost shape the vegetation of the *Campos de Altitude* (Safford, 1999b), the tree islands offer sites of reduced drought and frost through facilitation, hence allowing woody communities to exist above the tree line.

The “reproduction limitation hypothesis” could apply too, with the surrounding dense grasslands acting as an impenetrable barrier to tree seedling establishment, and reducing seedling survival through frost‐induced seedling mortality as previously observed for a forest–grassland matrix in the Western Indian Ghats (Joshi et al., 2019), while in tree islands seedling establishment could be promoted through facilitation. Frost occurs in Itatiaia on average 56 days per year at elevations of 2,200 m (Safford, 1999b), an elevation that broadly coincides with the location of the ten assessed tree islands.

### 4.3 Bamboo and tree islands

In line with our hypothesis, we found a tendency toward reduced functional and species diversity in tree islands with bamboo presence compared to tree islands without bamboo. Reduced Simpson diversity, which accounts for abundance and richness, hints that communities with bamboo presence have a less even and less rich species community. These differences in diversity could be due to prevalent disturbances, such as recurring fires (Aximoff & de Carvalho Rodrigues, 2011), promoting bamboo establishment, and impacting coexisting plants through competition and high flammability, consequently reducing species diversity (Pagad, 2016). This has been observed in understory communities in fragmented forests (Tomimatsu et al., 2011). Many of the sampled trees between 1 m and 1.5 m in our study are part of the understory and could be subjected to this process.

Decreases in functional and taxonomic diversity in tree islands with bamboo presence might result in lower resilience to environmental change due to biotic homogenization, that is, “the process by which species invasions and extinctions increase the genetic, taxonomic, or functional similarity of two or more locations over a specified time interval” (reviewed in Olden, 2008). Biotic homogenization could be promoted by future increases in fire frequency, because in the *Campos de Altitude* the bamboo *Chusquea pinifolia* has been shown to exhibit high resprouting ability after fire (Safford, 2001). This could result in changes of abundance and composition of co‐occurring woody communities toward poorer and uneven woody communities, for instance dominated by *M*. *gardneriana* and *M*. *umbellata*, the two most abundant species in tree islands with bamboo presence.

Bamboos can further impact co‐occurring plants by modifying the local hydrology (Fadrique et al., 2021; Pagad, 2016) and limiting water availability for co‐occurring trees (Takahashi et al., 2003). As observed in bamboo‐dominated Amazonian forests, selection favors drought‐tolerant trees due to competition for water imposed by bamboos (Fadrique et al., 2021). While we did not find any differences in CSR strategy between islands with and without bamboo, we found a trend toward lower CWM of FWU in woody communities with bamboo presence. For instance, this could result from bamboos promoting microclimatic sheltering and drought buffering for co‐occurring plants in a tree islands, hence minimizing water losses through transpiration and reducing the need for alternative water acquisition through FWU. It remains to be tested whether the biotic effect of bamboo on the woody communities is purely competitive or whether bamboo could also provide species‐specific facilitation for some life stages, as shown in montane temperate forests (Caccia et al., 2009).

Despite the trend of species diversity and functional diversity decreasing in response to bamboo presence, our study was limited by a relatively small sample size of 5 sites with bamboo presence and 5 without bamboo and we can hence only speculate on trends and patterns, but not on statistical power of the relationships. While many species of the genus *Chusquea* flower gregariously, observations suggested annual flowering of *Chusquea* in mountain environments (Clark, 1986), but the specific life‐history characteristics of the bamboo species *C*. *pinifolia* remain largely unknown. Hence, we cannot conclude with certainty on potential pathways of future invasion. A more comprehensive multiyear experimental study, including more plots, bamboo life‐history traits, establishment, and recruitment traits of woody species, as well as environmental variables, will be needed to elucidate on mechanisms underlying the facilitative and competitive interactions.

### 4.4 Climate change & the woody communities

The *Campos de Altitude* are particularly vulnerable to climate change due to their disjoint geographical distribution, restricted altitudinal range, high levels of endemism, and vegetation susceptibility to fires (Assis & de Mattos, 2016).

While there is a scarcity of climate change projections for Brazilian high elevation ecosystems (Scarano et al., 2016), regional climate change models of southeast Brazil project strong warming during the summer with maximum temperature increasing by 9°C and annual rainfall reductions of 40%–50% by the end of the century (Lyra et al., 2018). Moreover, tropical mountain systems are expected to warm disproportionately at higher elevations, such as shown for the Andes (Diaz et al., 2014). Alpine warming has often been associated with forest expansion and upward shift of tree lines which is locally modulated by adverse effects like topography, site history, and anthropogenic disturbance (Holtmeier & Broll, 2007). Isolated woody plants occurring in forest–grasslands ecotones in southern Brazil have been shown to promote tree seedlings from the forest, hence favoring forest expansion into grasslands (Mendoza‐Hernández et al., 2013) but also to provide safe sites for grassland species, as previously shown for tree islands in forest–tundra ecotones (Albertsen et al., 2014).

Contrary to the hypothetical forest expansion, the current trend of increased anthropogenic fire frequency (Aximoff & de Carvalho Rodrigues, 2011; Medina et al., 2016) might suppress forest and tree island expansion, and instead promote grass and shrub species with rapid vegetative regeneration, such as the bamboo *C*. *pinifolia* (Safford, 2001). Predicted intensification of droughts (Lyra et al., 2018) and reduction of fog occurrence in mountainous regions (Scarano et al., 2016) could particularly affect CS‐species because of water depletion and their high dependence upon water storage and uptake, as well as causing loss of S‐species and community originality if future droughts patterns exceed the tolerance limits of S‐species (Matos et al., 2020). Most species found in the tree islands fall in the first category with limited foliar water uptake (*M*. *gardneriana*, *M. umbellata*, and *B. stylosa*) and S‐selected species in the grasslands (e.g., *B*. *uncinella* and *P. trinervia*) could be lost, causing compositional and functional changes in the woody communities. The woody communities show more conservative water use and lower foliar water uptake than most grasses and shrubs in the *Campos de Altitude* (Matos et al., 2020). Hence, future droughts and less fog occurrence might exceed water acquisition and storage mechanisms and push the woody communities to their hydraulic limits, especially for species with low foliar water uptake, low wood density, and associated low resistance to cavitation (such as *A*. *serratifolia*).

An assessment of drought survival mechanisms like drought deciduousness, photosynthetic stems, tolerance of low water potentials, and xylem vulnerability to cavitation will be needed to give a more species‐specific picture of ecophysiological mechanisms in response to climate change in the woody communities.

## 5 CONCLUSION

With a lack of research on mountain biodiversity in Brazil (Martinelli, 2007), this study provides knowledge about the critically understudied montane tree islands. The prevalence of a woody community increases overall species and functional diversity of the *Campos de Altitude* ecosystem. The tree islands are ecophysiologically, structurally, and compositionally different from the grasslands, and interspersed trees within, in the *Campos de Altitude* yet show similarities in their stress‐tolerant strategy.

Our results point toward large‐scale environmental abiotic filtering shaping community strategy, and small‐scale biotic interactions, such as facilitation, to drive differences in CSR strategies between woody communities inside and outside the tree islands. Biotic processes such as bamboo presence also resulted in modified ecophysiology and in reduced species diversity.

The ecosystem deserves a closer investigation of facilitative interactions, as a potential mechanism promoting woody community persistence at these high altitudes. Experimental introduction of species inside and outside the tree islands could help elucidate the effect of facilitation onto woody plant establishment and trait selection to predict expansion or retreat the islands into the grasslands. Experimental treatment studies with bamboo, fire, and herbivory could be useful to characterize tree island resilience to biotic disturbance, while studies on ecophysiological traits related to drought tolerance could help to accurately model woody community response to drought intensification.

This study provides descriptive insights into the functional ecology of the woody communities and we hope that further trait‐based studies will help to elucidate management consequences allowing for synergies between the *Campos de Altitude* and the woody communities in a changing climate.

## CONFLICT OF INTEREST

The authors have no conflicts of interest to declare.

## AUTHOR CONTRIBUTIONS

**Tina Christmann:** Conceptualization (lead); Data curation (lead); Formal analysis (lead); Funding acquisition (lead); Investigation (lead); Methodology (equal); Project administration (lead); Visualization (equal); Writing‐original draft (lead); Writing‐review & editing (lead). **Bruno H. P. Rosado:** Conceptualization (equal); Formal analysis (supporting); Funding acquisition (supporting); Methodology (equal); Project administration (supporting); Supervision (supporting); Writing‐original draft (supporting); Writing‐review & editing (supporting). **Guillaume Delhaye:** Formal analysis (supporting); Methodology (supporting); Visualization (supporting); Writing‐original draft (supporting); Writing‐review & editing (supporting). **Ilaíne S. Matos:** Formal analysis (supporting); Methodology (supporting); Visualization (supporting); Writing‐original draft (supporting); Writing‐review & editing (supporting). **Helena L. Roland:** Investigation (equal); Methodology (supporting); Writing‐review & editing (supporting). **Yan C. Moraes:** Investigation (equal); Methodology (supporting); Writing‐review & editing (supporting). **Julia S. Drummond:** Investigation (equal); Methodology (supporting); Writing‐review & editing (supporting). **Imma Oliveras Menor:** Conceptualization (equal); Formal analysis (supporting); Funding acquisition (supporting); Investigation (supporting); Methodology (equal); Project administration (supporting); Supervision (lead); Visualization (supporting); Writing‐original draft (supporting); Writing‐review & editing (supporting).

## Supporting information

Supplementary Material

## Data Availability

Data and R‐scripts are publicly available on Dryad: https://doi.org/10.5061/dryad.gtht76hmk.
