# Supplementary material for: Functional assembly of tropical montane tree islands in the Atlantic Forest is shaped by stress tolerance, bamboo presence, and facilitation
Source: Ecol Evol. 2021 Jul 2;11(15):10164–77. doi: 10.1002/ece3.7824 (PMC8328411; doi:10.1002/ece3.7824)
Supplement: Supplementary file 1 — Supplementary Material [file ECE3-11-10164-s001.docx]

## **Supplementary material**

## **Functional assembly of tropical montane tree islands in the Atlantic forest is shaped by stress-tolerance, bamboo-invasion and facilitation**

*Christmann, Tina^1*^; Rosado, Bruno H. P.^2^; Delhaye, Guillaume^1^; Matos, Ilaíne S.^3^; Drummond, Julia S.^2^; Roland, Helena L.^2^; Moraes, Yan C.^2^; Oliveras, Imma^1*^*

Table S1:Metadata for the 10 forest islands, coordinates, and elevations and terrain values calculated from the SRTM-DEM, tree island size approximately measured on Google Earth and comments

| Tree island | GPS coordinates in S and W | | | inclination from S to N | Bamboo | Mean canopy height inside tree island [m] | Mean tree height [m] | Aspect [°] | Slope [%] | Ruggedness | Elevation  (DEM) | Elevation  (GPS) | Size in ha | comments |
| --- | --- | --- | --- | --- | --- | --- | --- | --- | --- | --- | --- | --- | --- | --- |
|  | **0m** | **10m** | 20m |  |  |  |  |  |  |  |  |  |  |  |
| A | 22.36702, 44.69806 | 22.36692, 44.6812 | 22.36683, 44.6817 | flat | B | 3.46 | 4.57 | 235.7 | 13.29 | 24.49 | 2434 | 2506 | 0.16 | very sharp forest edge |
| B | 22.3739, 44.70491 | 22.37379, 44.70514 | 22.37371, 44.70519 | down | N | 2.94 | 2.07 | 305 | 12.55 | 18.8 | 2450 | 2445 | 0.28 | a lot of re-sprouting |
| C | 22.37926, 44.69212 | 22.37919, 44.69217 | 22.3791, 44.69219 | down | N | 3.27 | 2.56 | 27.26 | 9.35 | 13.56 | 2425 | 2429 | 0.35 | very sharp forest edge, close to D and E |
| D | 22.38036, 44.69197 | 22.38029, 44.69213 | 22.38019, 44.69216 | down | B | 2.56 | 2.18 | 55.06 | 21.55 | 27.76 | 2439 | 2437 | 0.79 | close to C and E |
| E | 22.37944, 44.69156 | 22.3794, 44.6916 | 22.3793, 44.69161 | down | N | 2.38 | 1.74 | 118.5 | 10.01 | 13.15 | 2416 | 2421 | 0.11 | close to C and D |
| F | 22.37374, 44.70669 | 22.37367, 44.70671 | 22.37356, 44.70672 | concave | N | 4.27 | 2.67 | 10.22 | 15.37 | 19.46 | 2414 | 2413 | 0.41 | very small patch, beginning of transect next to road |
| G | 22.37479, 44.69845 | 22.37487, 44.69842 | 22.37495, 44.684 | steep up | B | 3.44 | 4.33 | 201.8 | 13.42 | 16.73 | 2438 | 2430 | 0.85 | big boulders, dense bamboo cover outside edge |
| H | 22.36994, 44.70793 | 22.36984, 44.70794 | 22.36986, 44.70802 | steep up | B | 5.86 | 2.08 | 222.31 | 21.9 | 27.78 | 2386 | 2396 | 1.48 | a lot of bamboo |
| I | 22.38716, 44.67455 | 22.38703, 44.67481 | 22.38721, 44.67484 | steep up | B | 5.59 | 2.21 | 171.9 | 26.77 | 35.7 | 2344 | 2322 | 0.39 | next to river and waterfall, big rock and hole halfway in transect |
| J | 22.37252, 44.70388 | 22.37261,44.7039 | 22.37258, 44.7039 | down | N | 5.0 | 3.05 | 333.4 | 5.66 | 8.77 | 2446 | 2442 | 0.42 | very small patch, beginning of transect next to road, from 15m onwards only Cortaderia, transect thus only 15m long |

**Supplementary methods 1:**

**Measurement of morphological traits (following protocols of Pérez-Harguindeguy and others 2013)**

For every tagged individual (188 plants), one sun-exposed branch with mature leaves was collected and stored for maximum 2h in wet black bags during transport and then rehydrated in for 3h (1.5h without cover and 1.5h with a black cover). Leaf morphological trait measurements were carried out on the same day and leaf water uptake on the next day. For WD, a 5-8cm piece of the branch, about 1cm thick, was debarked then submerged in water for 30min until saturation. After gentle drying with a tissue its volume was measured through water displacement method. After 72h drying at t 101–105°C to constant weight, WD was calculated as the ration between dry mass and fresh volume. For morphological leaf trait measurements, fresh mass of five mature leaves were collected from the rehydrated branches. Leaf area was measured on fresh leaves using ImageJ software based on a contrast threshold method. LT was measured with a calliper, on 5 fresh leaves e mid-point of the leaves and then averaged to obtain values per leave. Fresh mass of the five leaves was obtained using a precision scale (model …), then the leaves were oven-dried for 72 h and dry weight was obtained. Averages per leaf were then calculated. SLA was then calculated as the leaf area/fresh mass and LDMC as the oven dry mass/fresh mass.

**Supplementary methods 2:**

**LWU measurement procedure (following protocol by (Limm and others , 2009)**

The individual leaves were weighted to obtain the saturated leaf mass (Mass0) and area. After a 3h-drying period, each leaf was scanned and weighted (Mass1). The leaf petiole was sealed with vaseline and attached with a needle to a cup wall and the leaf lamina submerged in distilled water for 3h covered with dark plastic bag. After gently drying of leaf with paper towel, submersed leaf mass (Mass2) was obtained. For correction measurements, the leaf was left to re-dry for 5mins, then weighted (Mass3), hen again re-submersion of leaf for 1sec in water, gently dried and re-weighted (Mass4). Leaves were then stored in paper envelopes until 72h- drying in oven. We calculated the increase in leaf water content (LWU [%]) due to leaf water uptake for each submerged leaf with the following equation:

$$LWU=[\frac{Mass2-\left( Mass4-Mass3 \right)-MassDry}{\left( Mass1-MassDry \right)}-1]\cdot100\%$$

where, MassDry is the dry mass of the leaf measured after drying at 60°C for 72 h.

**Supplementary methods 3: CWM**

Community weighted means (CWM) were further calculated with the R-package ‘FD’, (Laliberté et al. 2015) for each subcommunity (10 inside sub communities) and outside (10 outside sub communities) weighing by species abundance and including intraspecific variation of the species between subcommunities (i.e. with trait means of each species in each subcommunity, as opposed to species means across all subcommunities).

**Supplementary methods 4: Variation partitioning**

Following Oliveras et al. (2020), we performed a variation partitioning analysis with the R-package ‘lme4’ (Bates et al. 2015), to examine trait variation in response to species-identity, inside vs outside and in response to differences between plots (=the 10 woody communities) with a linear mixed effect model. The model was set up across three ecological scales with three random factors (between species, between woody plants in tree islands and in the grasslands, nested within each plot).

$$lmer(trait \sim(1|Species) + (1|Plot/ inside\_outside)$$

Explained variances were extracted for each predictor. The residual variance included intra-specific variation and unknown random effects.

Table S2: Mean and standard error for traits and CSR strategy of the dominant species, including information on abundance and location of presence, and CWM for the entire woody community

| **Species** | **SLA [cm^2^/g]** | | **LA [cm^2^]** | | **LDMC [mg/g]** | | **LT [mm]** | | **WD [g/cm^3^]** | | **FWU [%]** | | **C [%]** | | **S [%]** | | **R [%]** | | **abundance [%]** | **Present inside or outside tree islands?** |
| --- | --- | --- | --- | --- | --- | --- | --- | --- | --- | --- | --- | --- | --- | --- | --- | --- | --- | --- | --- | --- |
| *Baccharis stylosa* | 16.21 | ±0.801 | 10.59 | ±1.22 | 410 | ±24 | 0.27 | ±0.018 | 0.95 | ±0.107 | 13.14 | ±1.68 | 23.73 | ±1.69 | 75.64 | ±1.77 | 0.64 | ±0.317 | 13.53 | both |
| *Baccharis uncinella* | 14.45 | ±0.589 | 0.41 | ±0.0198 | 500 | ±42.7 | 0.29 | ±0.016 | 0.61 | ±0.072 | 60.36 | ±11.1 | 1.40 | ±0.203 | 98.60 | ±0.203 | 0.00 | ±0 | 4.12 | out |
| *Archibaccharis serratifolia* | 23.82 | ±1.3 | 9.98 | ±1.05 | 340 | ±10 | 0.22 | ±0.018 | 0.57 | ±0.068 | 20.86 | ±2.06 | 25.92 | ±1.36 | 66.12 | ±2.74 | 7.96 | ±2.07 | 8.82 | both |
| *Symplocos (morpho-species)* | 14.68 | ±0.952 | 3.73 | ±0.819 | 400 | ±10 | 0.30 | ±0.028 | 1.09 | ±0.2 | 10.66 | ±3.77 | 14.24 | ±2.19 | 85.76 | ±2.2 | 0.00 | ±0. | 4.71 | both |
| *Gaultheria serrata* | 13.47 | ±0.722 | 9.09 | ±0.613 | 450 | ±46 | 0.39 | ±0.059 | 0.80 | ±0.109 | 9.52 | ±0.835 | 21.87 | ±1.15 | 78.13 | ±1.15 | 0.00 | ±0 | 5.29 | out |
| *Myrsine gadneriana* | 15.82 | ±0.822 | 18.46 | ±1.24 | 390 | 13 | 0.35 | ±0.014 | 0.92 | ±0.059 | 12.01 | ±1.45 | 31.87 | ±1.02 | 66.38 | ±1.45 | 1.75 | ±0.848 | 27.06 | both |
| *Myrsine umbellata* | 20.12 | ±1.17 | 13.15 | ±1.8 | 330 | ±9 | 0.30 | ±0.029 | 0.89 | ±0.079 | 4.56 | ±0.711 | 31.67 | ±1.72 | 63.97 | ±2.83 | 4.36 | ±1.62 | 8.24 | both |
| *Pleroma trinervia* | 19.92 | ±0.814 | 2.63 | ±0.597 | 340 | ±29 | 0.24 | ±0.017 | 0.62 | ±0.045 | 25.14 | ±2.95 | 10.37 | ±1.67 | 86.61 | ±2.07 | 3.02 | ±1.11 | 8.24 | both |
| ***Community weighted mean*** | 17.2 |  | 10.60 |  | 390 |  | 0.31 |  | 0.88 |  | 16.41 |  | 22.78 |  | 75.09 |  | 2.13 |  | total species number: 24 | |


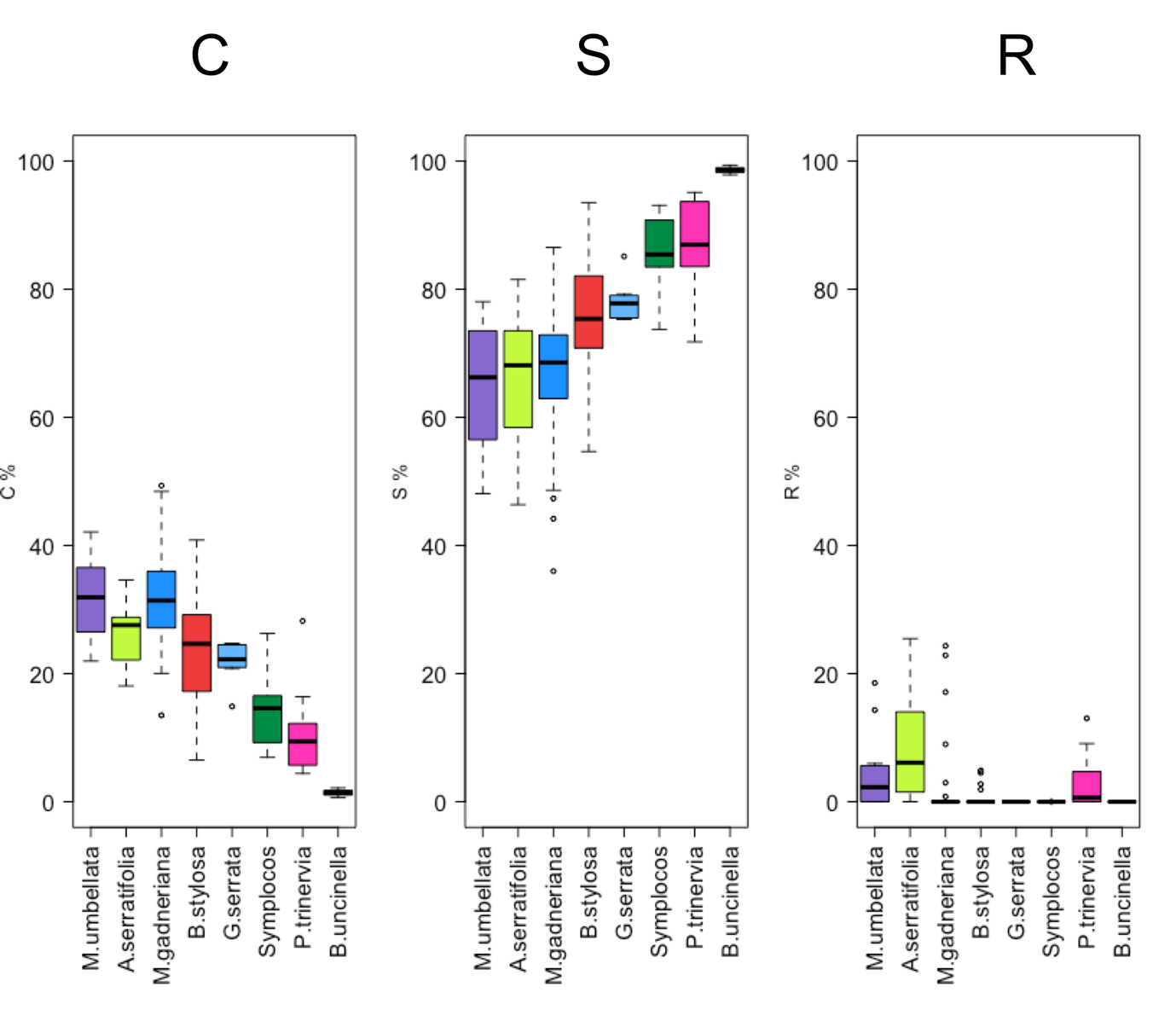


Figure S1: CSR strategy for the eight ‘dominant’ species, sorted after medians in S-strategy (n=136 individuals)

|  | **Trait comparison woody plants inside vs outside tree islands** | | | | | | | | | **Diversity comparison inside vs outside subcommunities** | | |
| --- | --- | --- | --- | --- | --- | --- | --- | --- | --- | --- | --- | --- |
|  | **SLA [cm^2^/g]** | **LA [cm^2^]** | **LDMC [mg/g]]** | **LT [mm]** | **WD [g/cm^3^]** | **FWU [%]** | **C [%]** | **S [%]** | **R [%]** | **Simpson index** | **Number species** | **FDis** |
| **Mean of CWM inside Islands** | 17.574 | 11.762 | 378.7 | 0.3137 | 0.9912 | 15.17 | 24.72 | 72.73 | 2.55 | 0.65 | 5.4 | 1.79 |
| **Mean of CWM outside Islands** | 15.77 | 7.297 | 425 | 0.29 | 0.804 | 26.612 | 16.381 | 82.69 | 00.925 | 0.48 | 4.1 | NA |
| **MW-test of CWM inside vs outside (p-value)** | **0.029** | **0.089** | 0.011 | 0.529 | 0.19 | 0.481 | **0.043** | **0.029** | **0.082** | 0.256 | 0.058 | NA |
|  | **Trait comparison woody plants in bamboo invaded and non- invaded islands** | | | | | | | | | **Diversity comparison bamboo invaded vs non invaded subcommunities** | | |
| **Mean CWM islands with bamboo** | 16.49 | 12.104 | 372 | 0.335 | 0.928 | 11.523 | 25.42 | 73.18 | 1.4 | 0.568 | 4.6 | 1.5 |
| **Mean CWM islands without bamboo** | 18.66 | 11.42 | 385 | 0.293 | 1.055 | 15.05 | 24.02 | 72.28 | 3.703 | 0.742 | 6.2 | 2.007 |
| **MW test of CWM bamboo vs non-bamboo (p-value)** | 0.222 | 1 | 0.69 | 0.151 | 0.841 | 0.056 | 0.421 | 1 | 0151 | **0.016** | 0.053 | **0.066** |

Table S3: Mean statistics and significance tests (Mann-Witney test) for differences in CWM of subcommunities inside and outside (top) and of tree island subcommunities with bamboo-presence and with no bamboo (bottom).

Table S4: List of species and their families

| **Species** | **Family** |
| --- | --- |
| Escallonia laevis*(Vell.) Sleumer* | Escaloniacea |
| *Baccharis grandimucronata Malag.* | Asteracea |
| Baccharis stylosa*Gardner* | Asteracea |
| Roupala montana*Aubl* | Proteacea |
| *Baccharis uncinella D.C.* | Asteracea |
| *Myrsine gardneriana A. DC* | Primulaceae |
| Myrceugenia alpigena*(DC.) Landrum* | Myrtaceae |
| Lepechinia speciosa*(A.St.-Hil. ex Benth.) Epling* | Lamiaceae |
| *Pleroma trinervia* | Melastomaceae |
| Leandra quinquedentata*Cogn* | Melastomaceae |
| Gaultheria serrata*(Vell.) Kin.-Gouv. ex Luteyn* | Ericaceae |
| *Baccharis altimontana (G. Heiden et al)* | Asteracea |
| *Baccharis brevifolia DC.* | Asteracea |
| *Baccharis glaziovii Baker* | Asteracea |
| *Baccharis platypoda D.C.* | Asteraceae |
| *Berberis laurina Thunb.* | Berberidaceae |
| *Myrsine umbellata Mart.* | Myrsinaceae |
| *Archibaccharis serratifolia (Kunth) S.F.Blake* | Asteraceae |
| **Morphospecies** | |
| Baccharis spp | Asteraceae |
| Berberis spp | Berberidaceae |
| Roupala spp | Proteaceae |
| Symplocos spp | Symplocaceae |
| Myrtaceae spp | Myrtaceae |
| Asteraceae spp | Asteraceae |
